# earlyMYCO: A Pilot Mother-Child Cohort Study to Assess Early-Life Exposure to Mycotoxins—Challenges and Lessons Learned

**DOI:** 10.3390/ijerph19137716

**Published:** 2022-06-23

**Authors:** Carla Martins, Ricardo Assunção, Ana Costa, Débora Serrano, Lia Visintin, Marthe De Boevre, Carl Lachat, Arnau Vidal, Sarah De Saeger, Sónia Namorado, Cristina Vidigal, Elisabete Almeida, Paula Alvito, Carla Nunes

**Affiliations:** 1 National Institute of Health Dr Ricardo Jorge, Avenida Padre Cruz, 1649-016 Lisbon, Portugal; rassuncao@egasmoniz.edu.pt (R.A.); sonia.namorado@insa.min-saude.pt (S.N.); paula.alvito@insa.min-saude.pt (P.A.); 2 Centre for Environmental and Marine Studies, Campus Universitário de Santiago, University of Aveiro, 3810-193 Aveiro, Portugal; ana.costa@ensp.unl.pt (A.C.); debora.serrano@ensp.unl.pt (D.S.); 3 Public Health Research Centre, NOVA National School of Public Health, Universidade NOVA de Lisboa, Avenida Padre Cruz, 1600-560 Lisbon, Portugal; cnunes@ensp.unl.pt; 4 Comprehensive Health Research Center, Campo Mártires da Pátria, Universidade NOVA de Lisboa, 1169-056 Lisbon, Portugal; 5 Instituto Universitário Egas Moniz (IUEM), Egas Moniz-Cooperativa de Ensino Superior, CRL, 2829-511 Caparica, Portugal; 6 Centre of Excellence in Mycotoxicology and Public Health, Faculty of Pharmaceutical Sciences, Ghent University, Ottergemsesteenweg 460, B-9000 Ghent, Belgium; lia.visintin@ugent.be (L.V.); marthe.deboevre@ugent.be (M.D.B.); carl.lachat@ugent.be (C.L.); arnauvidalcorominas@gmail.com (A.V.); sarah.desaeger@ugent.be (S.D.S.); 7 ACES Lisboa Central, Regional Health Administration of Lisbon and Tagus Valley (ARSLVT), 1700-179 Lisbon, Portugal; cristina.vidigal@arslvt.min-saude.pt (C.V.); elisabete.lamy@arslvt.min-saude.pt (E.A.)

**Keywords:** early-life exposure, birth cohorts, mycotoxins, exposome, Portugal

## Abstract

Early-life exposure occurs during gestation through transfer to the fetus and later, during lactation. Recent monitoring data revealed that the Portuguese population is exposed to mycotoxins, including young children. This study aimed to develop a pilot study to assess the early-life exposure to mycotoxins through a mother–child cohort, and to identify the associated challenges. Participants were recruited during pregnancy (1st trimester) and followed-up in three moments of observation: 2nd trimester of pregnancy (mother), and 1st and 6th month of the child’s life (mother and child), with the collection of biological samples and sociodemographic and food consumption data. The earlyMYCO pilot study enrolled 19 mother–child pairs. The analysis of biological samples from participants revealed the presence of 4 out of 15 and 5 out of 18 mycotoxins’ biomarkers of exposure in urine and breast milk samples, respectively. The main aspects identified as contributors for the successful development of the cohort were the multidisciplinary and dedicated team members in healthcare units, reduced burden of participation, and the availability of healthcare units for the implementation of the fieldwork. Challenges faced, lessons learned, and suggestions were discussed as a contribution for the development of further studies in this area.

## 1. Introduction

The first period of life encompasses a critical window for human health and development. The exposure to hazardous chemicals during this critical period has been linked to an increased risk for several human health disorders [1]. In utero exposure to external stressors during critical windows can interfere with developmental processes and permanently alter body structure, metabolism and physiology, leading to chronic pathologies later in life [2].

The exposome, a concept coined in 2005 by Christopher Wild, is related with “all exposures from conception onward, including those from lifestyle, diet and the environment” [3]. The food exposome, i.e., the totality of individual exposures from food, is a major contribution to the individual external exposures. Food in this context, may include not only nutrients, but also contaminants that represent a potential health threat [4,5]. Food chemical contaminants such as mycotoxins are well-known compounds exerting carcinogenic, nephrotoxic, hepatotoxic, estrogenic and immunosuppressive properties [6,7,8,9]. Due to their prevalence in food commodities, mycotoxins adversely affect health [10]. Furthermore, the transfer of mycotoxins to the fetus through the placental barrier and to newborns through breastfeeding was reported by a number of studies [11,12,13,14], reflecting the increasing interest of the scientific community on this topic.

Biomonitoring studies have shown to be of increasing importance in the last years to assess the human exposure to mycotoxins [15,16] proving to be an alternative to the exposure assessment through combination of mycotoxin occurrence and food consumption data [17,18,19]. Mycotoxin human biomonitoring data in vulnerable populations, such as pregnant women and infants, are scarce and specific efforts are needed to assess maternal dietary exposure, blood plasma and breast milk levels of mycotoxins. Perinatal epidemiology allows a comprehensive analysis of mother-and-child pair exposure to mycotoxins and is considered adequate for researching the exposome [20,21]. Additionally, the development of birth cohorts offers the possibility of a prospective follow up of participants, being particularly important to examine the effects of early-life external stressors on growth, development, and long-term health [22].

**Figure 1:**
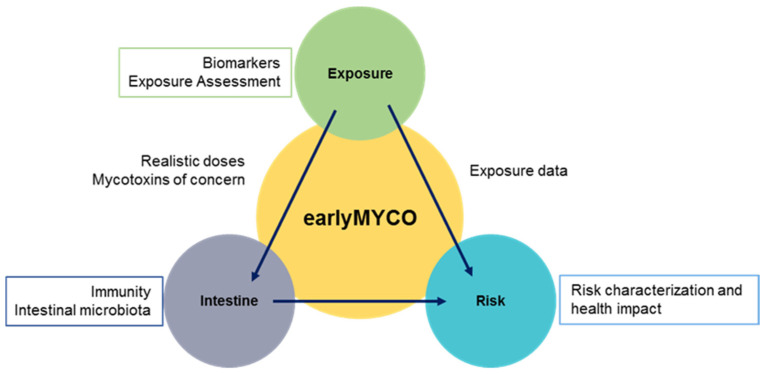
Conceptual framework of earlyMYCO project.

Recent studies reported that Portuguese children up to 3 years old are exposed to multiple mycotoxins [23,24,25,26,27], which may constitute a potential health concern. Human biomonitoring studies confirmed the exposure of Portuguese adult population to several mycotoxins [28,29]. Considering the potential impact in health and the scarce data available regarding early-life exposure to mycotoxins, a new project named earlyMYCO (early-life exposure to MYCOtoxins and its impact on health) was developed and implemented. The project considered three major domains: epidemiology, toxicology, and risk assessment, hypothesizing that early-life exposure to mycotoxins could earlyMYCO—a pilot mother–child cohort study to assess early-life exposure to mycotoxins impact children’s and future adults’ health (Figure 1). To accomplish the epidemiological approach, a pilot cohort study was developed to assess the exposure of pregnant women and infants to mycotoxins, including the collection of biological samples, sociodemographic and food consumption data. Further studies on the remaining domains will be detailed elsewhere. The design of the earlyMYCO cohort was based on several published studies [30,31,32,33,34].

The present manuscript aims to describe the design of a mother-and-child prospective cohort pilot study performed within the earlyMYCO project in the region of Lisbon, Portugal. The challenges faced and lessons learned during the implementation and development are discussed, as a main outcome for future studies on this scientific research domain.

## 2. Materials and Methods

### 2.1. Study Plan, Design and Participants

The earlyMYCO pilot study was carefully planned by a multidisciplinary team (biomedical scientists, nurses, medical doctors, epidemiologists, and other researchers), who were further involved in the fieldwork, namely on the following tasks: recruitment and contacts with the participants, collection of biological samples, application of questionnaires, and transport and storage of biological samples. Considering the aim of assessing early-life exposure to mycotoxins, a prospective cohort study was planned based on previous studies dedicated to assessing exposure to mycotoxins and/or environmental contaminants in pregnancy and early ages. The studies contributing the most to the design of earlyMYCO pilot study are summarized in Table S1, concerning the characteristics related with design, participants, follow-up moments, data instruments and biological samples collection.

The target population included all mother–child pairs, enrolled as patients in primary care health units (UCSP). During the year 2017, each UCSP provided maternal healthcare to 300–400 pregnant women. The inclusion criteria are the following: (i) being pregnant, (ii) aged above 18 years old, (iii) be a patient at UCSP, (iv) be able to understand the earlyMYCO’s documents in Portuguese, and (v) be able to attend the interviews and participate in the procedures for the collection of the biological samples, i.e., blood, urine and breast milk. Women that fulfill all the inclusion criteria of the study were considered as eligible for the earlyMYCO study. The women were considered as ‘unknown eligible’ if the following situations occurred: (i) be unable to establish a telephonic contact and (ii) to withdraw from the study before being possible to collect any information to validate the eligibility criteria.

An eligible woman was considered as a non-participant if she did not participate in the different moments of the earlyMYCO, did not meet one of the participation criteria or did not attend the first moment of observation. If a participant withdrew from the study, did not provide blood samples, had a pregnancy loss, or did not attend the interviews in at least two sequential moments of the study, it was considered a loss to follow-up. The fieldwork of earlyMYCO pilot study was conducted in the Lisbon municipality with the participation of two UCSP belonging to the Lisbon Central Primary Health Care Group, between October 2019 and December 2020.

### 2.2. Recruitment Strategy, Study Instruments and Data Collection

**Figure 2:**
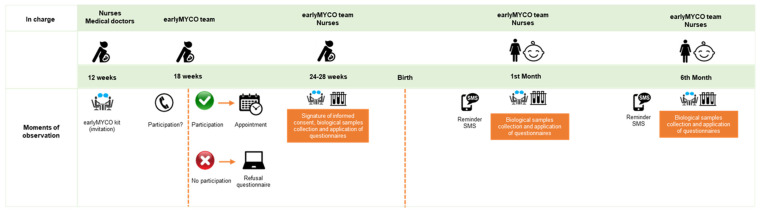
Design of earlyMYCO mother–child pilot cohort study.

Participants were contacted by medical doctors or nurses during the 12-weeks pregnancy medical appointments and received the earlyMYCO kit that included an invitation letter, an information leaflet about mycotoxins, detailed information about the earlyMYCO study, and the informed consent forms. Six weeks after the health professional contact, pregnant women were contacted by a member of the earlyMYCO team to determine the interest to collaborate in the study. The potential participant was encouraged to clarify any doubts about the study and the participant involvement. If the pregnant woman was not willing to participate in the study, a refusal questionnaire was applied to collect information about the reasons of non-participation (Figure 2). If the pregnant woman was willing to participate in the study, the first moment of observation was scheduled for 24–28 weeks of pregnancy, followed by the 2nd and 3rd moments of observation at the 1st and 6th month of child’s life, respectively. All three moments of observation included the application of sociodemographic and food consumption questionnaires and the collection of biological samples (Figure 2).

Regarding biological samples, a venipuncture procedure was performed by a biomedical scientist for the collection of serum and plasma samples at the 1st moment of observation. The participants (mother and child) were also asked to collect a spot urine sample in the three moments of observation. The collection of breast milk in the 2nd and 3rd moments of observation was performed by the mother, at home, in the morning.

The sociodemographic questionnaire included the following data: demographic (age, date of birth, country of birth, marital status); geographic (postal code, which allows classifying the zone of residency and urbanization); and socioeconomic (working status, level of education, family monthly income). The anthropometric questionnaire included the following data, where applicable: body weight, height, gender, gestation weeks.

For food consumption data, a 24 h recall and a Food Frequency Questionnaire (FFQ) were used to estimate consumption in the last 24 h before the moment of observation as well as the frequency of consumption of several food groups (dairy products, cereal-based products, fruits and dried fruits, smoked meat, meat, fish), respectively. A photographic manual developed under the National Food, Nutrition and Physical Activity Survey 2015–2016 (https://ian-af.up.pt/en, accessed on 2 December 2019) was used to enable a more accurate portion size reporting and estimation. Additionally, the number of times that the child was breastfed during the 24h before the questionnaire was recorded. If the child was fed with infant formula, the volume of each meal was registered. All questionnaires were previously developed and validated within the National Food, Nutrition and Physical Activity Survey (2015–2016) [35].

### 2.3. Ethics

Ethical approval was obtained from the Ethical Committees for Health of the National Institute of Health Doutor Ricardo Jorge and the Regional Health Administration of Lisbon and Tagus Valley. All participants provided their written informed consent according to the Ethical Principles for Medical Research involving human subjects expressed in the Declaration of Helsinki and the national legislation. The informed consent forms were signed during the first moment of observation and were separated for the mother and the child. The informed consent form for the child participation was signed by the parents or legal tutors. Data collection was performed under pseudo-anonymization.

### 2.4. Sample Analysis—Analytical Conditions

#### 2.4.1. Chemicals and Reagents

Mycotoxins standards for deoxynivalenol (DON) and ochratoxin A (OTA) were obtained from Merck (Darmstadt, Germany). Mycotoxin standards for aflatoxin B_1_ (AFB_1_), aflatoxin B_2_ (AFB_2_), aflatoxin G_1_ (AFG_1_), aflatoxin G_2_ (AFG_2_), aflatoxin M_1_ (AFM_1_), fumonisin B_1_ (FB_1_), fumonisin B_2_ (FB_2_), fumonisin B_3_ (FB_3_), nivalenol (NIV), zearalenone (ZEN), α-zearalenol (α-ZEL), β-zearalenol (β-ZEL), and hydrolysed fumonisin B_1_ (HFB_1_) were purchased from Fermentek. Mycotoxins standards for ochratoxin-α (OT-α), isotope-labeled internal standard ^13^C_20_ ochratoxin A (OTA-^13^C_20_), ^13^C_17_ aflatoxin B1 (AFB_1_-^13^C_17_), and ^13^C_15_ deoxynivalenol (DON-^13^C_15_) were obtained from Romer Labs^®^ (Tulln, Austria). Mycotoxin solid standards were dissolved in methanol or acetonitrile according to the respective certificate of analysis and were stored at −20 °C. The working solutions were prepared in methanol and stored at −20 °C. Ultrapure water was produced on a Milli-Q^®^ SP Reagent water system from Millipore Corp. (Brussels, Belgium). Methanol absolute LC-MS grade (MeOH), acetonitrile absolute LC-MS grade (ACN), formic acid (99%, HCOOH), and acetic acid (99.5%, CH_3_COOH) were purchased from BioSolve (Valkenswaard, The Netherlands). Sodium chloride (>99.9%, NaCl), magnesium sulphate anhydrous (99.3%, MgSO_4_), and N-hexane HiperSolv CHROMANORM were supplied by Avantor, VWR International (Radnor, PA, USA). Ammonium Acetate (≥98%, NH_4_CH_3_COO) was obtained from Merck (Darmstadt, Germany).

#### 2.4.2. Sample Preparation

Urine: A QuEChERS-based procedure (Quick, Easy, Cheap, Effective, Rugged, Safe) was used for the urine sample preparation. The method is according to [28,36]. Briefly, the urine samples were defrosted and allowed to acclimatize to room temperature. A volume of 2 mL of each sample was spiked with 20 μL of internal standards mixture and mixed with 18 mL of acetonitrile/water/formic acid (52/45/3, *v*/*v*/*v*) in a 50 mL centrifuge tube. Thereafter, 4 g of magnesium sulphate and 1 g of sodium chloride were added into the extraction tube and the mixture was vortex-shaken for 30 s. Samples were overhead-shaken for 30 min. After centrifugation (3000× *g*, 10 min), 7 mL of the organic layer was taken and evaporated to dryness under a gentle nitrogen flow at 40 °C. The residue obtained was dissolved in 250 µL of injection solvent (H_2_O/MeOH, 70/30, *v*/*v*) and centrifuged (4000× *g*, 7 min). Finally, 100 µL was transferred into a UPLC vial upon LC-MS/MS analysis.

Breast milk: A procedure based on double liquid–liquid extraction and a solid-phase extraction was used for the breastmilk sample preparation. Briefly, the breastmilk samples were defrosted and allowed to acclimatize to room temperature. A volume of 1 mL of each sample was spiked with 10 µL of the internal standard mixture in a 50 mL centrifuge tube, incubated in the dark for 15 min, and mixed with 4 mL of ACN/formic acid (99/1 *v*/*v*). Thereafter, the tubes were vortex-shaken for 30 s, overhead-shaken for 10 min, and centrifuged (3000× *g*, 10 min). The supernatants were transferred into the OASIS^®^ PRiME HLB 6cc (200 mg) Extraction Cartridges (Waters^®^, Manchester, UK) after conditioning with 3 mL ACN/Formic acid (99/1 *v*/*v*). The eluates were collected in test tubes and dried under a gentle nitrogen flow at 40 °C. Afterward, the dry residues were reconstituted in 200 μL of injection solvent (H_2_O/MeOH, 70/30, *v*/*v*) and vortex shaken for 1 min. Subsequently, 200 µL of n-hexane was added to the tubes, the samples were vortex-shaken again for 1 min, and centrifuged (3000× *g*, 5 min). Finally, the samples were transferred in a PVD Centrifugal Filter Units, Millipore^®^ filter 0.22 µm, ultracentrifuged (10,000 rpm, 10 min), and 100 µL of the lower phase of the filtrates was transferred into a UPLC vial upon LC-MS/MS analysis.

#### 2.4.3. Instrumental Analysis

Sample analysis was performed in a Waters^®^ Acquity UPLC system coupled to a Quattro XEVO TQ-S mass spectrometer (Waters^®^, Manchester, UK). The software used for data acquisition and processing was MassLynx™ version 4.1 and QuanLynx^®^ version 4.1 (Waters^®^, Manchester, UK). Separation of analytes was carried out by an HSS T-3 column (2.1 × 100 mm, 1.8 μm) (Waters^®^, Manchester, UK). The column temperature was maintained at 40 °C and the samples temperature was kept at 10 °C. The two different mobile phases used were: watery mobile phase A (H_2_O/MeOH/acetic acid; 94/5/1, *v*/*v*/*v*) and organic mobile phase B (MeOH/H_2_O /acetic acid; 97/2/1, *v*/*v*/*v*), both buffered with 5 mM NH_4_CH_3_COO. The flow rate was set at 0.3 mL/min. The total run time was 18 min. The volume of sample injected into the UPLC system was 10 μL. The gradient started at 95% A, decreasing to 35% A until 7 min, followed by a decrease to 25% A until 11 min. Mobile phase B immediately changed to 99% at 13 min, kept in the same conditions until 14 min, and immediately changed to initial conditions at 14.1 min (95% mobile phase A). The column was allowed to equilibrate until 18 min. The ESI-source was operated in positive mode (ESI+), with the capillary voltage set at 3.30 kV. Source and desolvation temperatures were 150 °C and 600 °C, respectively. Nitrogen was applied as desolvation and curtain gas. The desolvation flow was 1000 L/h, while the cone flow was 150 L/h. Argon was applied as collision gas, its pressure was 9 × 10^−6^ bar. All the analytes were previously tuned to determine specific fragmentations, dwell time, collision energy, and cone voltage to ensure accurate identification. The Multiple Reaction Monitoring (MRM) table contains all the transitions and parameters optimized for the analytes of interest (Table S2).

#### 2.4.4. Validation Experiments

An in-house validation was conducted following EU Commission Decision 2002/657/EC [37] and according to IUPAC guidelines [38]. The parameters investigated included limit of detection (LOD), limit of quantification (LOQ), intra and inter-day precision expressed as relative standard deviation (RSD), apparent recovery, and linearity. The validation process consisted of preparation and analysis of three matrix-matched calibration curve replicates during three different working days, for a total of 9 calibrations. The calibration plots were built plotting the analyte areas corrected by the area of the internal standard against the concentration. Evaluating the linearity, the homogeneity of variance was checked before fitting the linear model. The linearity was interpreted graphically using a scatter plot. Limit of detection (LOD) was calculated as three times the standard error of the intercept, divided by the slope of the standard curve; the limit of quantification (LOQ) was similar, differing by six times the standard error. The calculated LOD and LOQ were also verified by the signal-to-noise ratio (s/n) (Tables S3 and S4).

#### 2.4.5. Data Analysis

A descriptive analysis included tabulation of frequencies for categorical variables and classic descriptive measures for numerical variables (minimum, maximum, median, mean, standard deviation, where appropriate). Samples were considered positive if mycotoxins’ biomarkers concentrations were higher than the respective LOQ. These analyses were performed in Microsoft Excel 2016 (Microsoft Corporation, Redmond, WA, USA).

## 3. Results

### 3.1. Response Rates and Collected Data

Regarding the eligibility criteria, 29 women were registered by the medical doctors and/or nurses to participate and to whom the earlyMYCO kit was delivered. After the first telephone call to confirm their availability for the study, five women refused to participate due to lack of time (n = 4) and due to the involvement of the newborn (n = 1), representing a refusal rate of 17.2%. Due to several constraints associated with COVID-19 pandemic with total closure of primary health care units during the first lockdown (March–June 2020) and with severe limitations afterwards (June 2020–December 2020), five participants did not start their participation in the pilot study, although being available and agreeing to participate. The earlyMYCO pilot study enrolled 19 pairs of mother and children as participants. For the 2nd and 3rd moments of observation, nine and eight participants (mother and child pairs) remained enrolled in the study, respectively. Thus, the loss to follow-up associated with this pilot study was determined as follows: 9/19 (47%) from the 1st to the 2nd moment of observation, and 1/9 (11%) from the 2nd to the 3rd moment of observation.

During the three moments of observation, biological samples were collected by the earlyMYCO team and the participants, as well as data regarding food consumption (Table S5). In the 1st moment of observation, when members of the earlyMYCO team performed all the biological sample collection and interviews, all questionnaires were filled with no missing information. Regarding biological samples, one participant (1/19) accepted to participate in the study but refused to collect blood. During the 2nd and 3rd moments of observation, and due to the lockdowns and/or restrictions imposed by the COVID-19 pandemic, participants self-collected biological samples and self-filled the food consumption questionnaire (24 h dietary recall), with the support of a photographic manual. In the 2nd moment of observation, all biological samples and food questionnaires were collected and filled, respectively. During the 3rd moment of observation, one participant did not collect child’s urine (1/8), three participants did not fill the 24 h dietary recall (3/8) and one participant did not fill completely the 24 h dietary recall (1/8) (Table S5).

### 3.2. Sociodemographic, Mycotoxins’ Biomarkers and Food Consumption Data

Table 1 presents the characterization of participants (mothers) regarding the sociodemographic and anthropometric variables, at the 1st moment of observation.

Participants in the earlyMYCO pilot study (n = 19) were mainly born in Portugal (47.4%) and Brazil (36.8%) and were aged 24–49 years, with a median age of 34 years. The participation in earlyMYCO pilot study was initiated between 24 and 24 weeks of gestation. Regarding gestational age at the time of delivery, all infants were full-term born between 38 and 41 weeks of pregnancy.

Table 2 presents the results of mycotoxin biomarkers of exposure determined in the biological samples (urine and breast milk) of the 19 mother–child pairs. The analysis of biological samples from participants revealed the presence of four out of 15 and five out of 18 mycotoxins’ biomarkers of exposure in urine and breast milk samples, respectively. AFM_1_, AFB_2_, AFG_1_, AFG_2_, AFL, NIV, FB_3_, HFB_1_ and STER were not detected in any of the analyzed samples. The analysis of biological samples allowed to quantify three out of 15 (AFB_1_, OTA, DON) and three out of 18 (AFB_1_, aZEL, FB_1_) mycotoxins’ biomarkers of exposure in urine and breast milk samples, respectively. FB_2_ and FB_3_ in breast milk and bZEL in urine were found in levels above the LOD and below the LOQ of the respective analytical methods.

Regarding urine samples of the mothers, 2/19 samples for the 1st moment of observation were found positive for mycotoxins’ biomarkers (>LOQ). Urine samples collected during the 2nd and 3rd moments of observation were negative for all mycotoxins’ biomarkers or presented biomarkers in levels between the instrumental limits (DON and b-ZEL). Regarding breast milk samples, 2/7 samples were found positive for mycotoxins’ biomarkers (>LOQ) for the 2nd moment of observation. Breast milk samples collected during the 3rd moments of observation presented biomarkers in levels between the instrumental limits (AFB_1_, FB_1_, FB_2_ and FB_3_). For the children urine samples, 2/9 and 1/7 samples were found positive for mycotoxins’ biomarkers (>LOQ) for the 2nd and 3rd moments of observation, respectively. The mycotoxins’ biomarkers detected in samples were the following: AFB_1_, OTA and DON in urine samples (mother and children), and AFB1, aZEL and FB_1_ in breast milk samples.

Co-occurrence of mycotoxins was identified in three breast milk samples, with two different mixtures identified: FB_1_ and AFB_1_ (two samples), and aZEL, FB_1_ and AFB_1_ (one sample). Regarding urine samples, only one sample from a child presented simultaneously two mycotoxins, OTA and DON.

When considering results of mother and child paired-samples, DON was the only mycotoxin detected in the urine samples of one pair of participants (1/9) in the 2nd moment of observation and from an exclusively breastfed child, thus showing a possible lactational transfer of DON. The other mycotoxins were detected independently in biological samples (urine and breast milk) of mother and children, but with no relation within each other.

The food groups representing the dietary habits of the participants are presented in Table 3. From the FFQ it is possible to verify that foods consumed more frequently during the week are bread, dairy products, non-alcoholic drinks (tea and coffee), animal products (meat and fish) and pasta. These observations are confirmed by data obtained within the 24 h dietary recalls for the different moments of observation. Regarding infants, 2/9 were fed with infant formula (2nd moment of observation), 2/8 were fed with infant formula and vegetables (3rd moment of observation), and 7/9 and 6/8 were exclusively breastfed (2nd and 3rd moments of observation, respectively) (data not shown).

## 4. Discussion

Epidemiological studies with a robust and longitudinal design to assess the early-life exposure to mycotoxins and its potential health consequences for newborns, including later in life, are needed. The combination of epidemiological and toxicological approaches may strengthen the level of evidence of health and biological effects of early-life exposure [21]. De Ruyck et al. highlighted the importance of comparing biological fluids, such as urine and blood, to correctly elucidate a comprehensive cross-section of internal mycotoxin exposures [19]. These sampling characteristics contributed to reduce the uncertainty of estimates and are in accordance with the recommendations for generating data on intake. Further, biomarkers in blood and urine along with breast milk measures are adequate to better understand the toxicokinetic, including lactation transfer rates and to assess to what extent the breastfeeding is driving exposures in children [11,39].

Since this was a pilot study, these findings and lessons learned and discussed are suggested to be considered for the development of future studies. The development of a pilot study is always an important contribution for the implementation of a larger survey, allowing the refinement of methodologies and leading to the improvement of performance indicators [40]. An important contribution of this pilot study for the development of future studies is the capacity building obtained among researchers from different institutions with expertise in different and complementary scientific backgrounds, allowing a reflection herewith on some of the challenges associated and lessons learned with the development of fieldwork for these studies. Firstly, it is fundamental to have dedicated staff in the health units for the several phases of fieldwork (recruitment, sample collection, questionnaires application, results delivery) for a high compliance of participants and to prevent the loss to follow-up. Although nowadays it is possible to develop studies in web-based platforms and avoiding in-person interviews, there are some procedures and questionnaires that might be difficult for participants to fill and/or collect by themselves. The support of an interviewer will contribute to the quality, accuracy and completeness of data obtained during the moments of observation. This was verified during the present pilot study through the increase of missing and incomplete data in the 3rd moment of observation where the food consumption questionnaires were filled without support of the interviewer (Table S5). Regarding the loss to follow-up that should always be minimized to increase the robustness of the study, in the earlyMYCO pilot study several details were included to accomplish this issue: dedicated team for fieldwork, several reminders, contact personalization [41]. The highest loss to follow-up observed between the 1st and 2nd moments of observation was mainly due to the restrictions imposed by COVID-19 lockdowns. Between the 2nd and 3rd moments of observation the loss to follow-up was reduced, confirming that this issue was correctly addressed in the study design and that the efforts were effective. Secondly, an important issue is the minimization of the burden associated with the participation for the recruited individuals, being this especially important in birth cohorts since the participants are pregnant women and newborns and/or infants, whereas mothers will be responsible for the children’s participation and therefore loaded with the burden of participation [42]. The design of future studies should include time points coincident with programmed health checks (e.g., doctor/nurse appointments, vaccination) to avoid unnecessary travels to sampling facilities. The burden could also be reduced by previously supplying the participants with the sample containers, allowing collecting the biological samples at home when possible and feasible. Thirdly, the availability of the healthcare units is also fundamental, and it is important to emphasize that every event affecting the regular functioning of these units will have consequences on the development of these studies. The earlyMYCO pilot study faced an unexpected challenge related with the COVID-19 pandemic, with a total lockdown occurring during the years 2020–2021. Nevertheless, half of the participants maintained their availability to participate and agreed to collect the samples at home, with the earlyMYCO team ensuring the transport of samples to the laboratory. Considering this, it is advisable to design simultaneously a backup plan with alternative ways of performing the sample collection and data collection. Fourthly, the inclusion of additional variables in the sociodemographic, anthropometric and food consumption questionnaires would allow better characterizing the determinants of exposure and refining the statistical analysis. If possible, the inclusion of an additional timepoints for observation would also be desirable, especially in the first months of pregnancy (2–4 months) to better characterize the early gestational exposure. Finally, another pertinent aspect that should be discussed is the number of participants that is a crucial aspect of a survey to ensure the robustness and validity of results. Considering birth cohorts include participants in a vulnerable period of life (pregnant women and infants) and high refusal rates are possible, all efforts should be focused on increasing the number of participants. Some of the aspects related with the burden of participants were mentioned above, but others related with the active recruitment should also be referred to. The number of participant health units should be the highest as possible considering the resources allocated, with the desirable involvement of the private sector, and a dissemination plan should be implemented taking advantage of media (newspapers, TV programs or social media). All these issues should be considered when designing the sample collection and the follow-up moments to promote not only a high adherence to the study but also the availability to keep participants enrolled for the follow-up moments.

Recent data available for Portugal for the adult population referred the exposure to DON, ZEN, AFM_1_, AFB_1_, AFB_2_, AFG_1_, FB_1_, FB_2_, CIT, AOH and OTA [28,29]. Silva et al. also reported the exposure to OTA of children aged 2 to 13 years old, with 92.9% of positive samples and average concentration of 0.020 ng/mL [43]. Gratz et al. reported the presence of other mycotoxins (DON, NIV, ZEN, HT-2, T-2) besides OTA in urine samples from children aged above 2 years old in the United Kingdom, in a frequency ranging from 5% to 100% [44]. The frequency of positive samples in the present study is lower than those referred in the literature, but this could be justified by the instrumental limits of the applied analytical method. Additionally, the number of participants may also hamper the assessment of early-life exposure in this study. One important aspect that should be referred is the simultaneous detection of mycotoxins’ biomarkers in samples collected under the earlyMYCO study, thus showing exposure to different mycotoxins. Different combinations of mycotoxins’ biomarkers were found in breast milk samples (AFB_1_ + FB_1_ and AFB_1_ + aZEL + FB_1_) and in urine samples (OTA + DON). The co-occurrence of mycotoxins was also referred to previously in studies assessing early-life exposure mainly of aflatoxins, DON, OTA and ZEN [44,45,46]. A correlation between levels of mycotoxins in urine and breast milk was previously reported (r = 0.64) [46]. However, in the present study those findings were not possible to assess in the analyzed samples due to the number of participants enrolled. However, this issue should be considered in larger surveys with a higher number of participants.

## 5. Conclusions

To the best of our knowledge, few studies [31,33,45] were available considering different moments of observation to assess exposure to mycotoxins during early-life stages, with a study design considering the collection of several biological samples and food consumption data through different questionnaires. The complexity of birth cohorts implies a multidisciplinary team comprising a wide range of backgrounds, such as public health professionals, epidemiologists, statisticians, and biomedical researchers. The involvement of several institutions is also needed; healthcare units, laboratories, and academia should work together to fulfil the fieldwork requirements. In Portugal, despite growing evidence of harmful effects of mycotoxins, no national data on exposure of pregnant women and children were available until now, so this pilot study contributed to generate new data and confirmed early-life exposure to mycotoxins. Considering the lessons learned, additional and broader studies should be developed to assess the exposure of these vulnerable groups in Portugal, thus ensuring that the appropriate risk management measures are in place to protect vulnerable population groups’ health.

## Figures and Tables

**Table 1:** Characterization of participants (mothers) in the earlyMYCO pilot study (n = 19).

Sociodemographic		**n**	**%**	
	**Country of birth**			
	Portugal	9	47.4	
	Brazil	7	36.8	
	Republic of Cabo Verde	2	10.5	
	United Kingdom	1	5.3	
	**Education level**			
	10–12 years	7	36.8	
	>12 years	12	63.2	
	**Marital Status**			
	Single	4	21.1	
	Divorced	2	10.5	
	Married or living together	12	68.4	
	**Working condition**			
	Worker for remuneration or profit	18	94.7	
	Unemployed	0	0.0	
	Other *	1	5.3	
	**Household income** (€)			
	485–970 €	2	10.5	
	971–1455 €	3	15.8	
	1456–1940 €	2	10.5	
	More than 1941 €	13	42.2	
	Do not know/answered	4	21.1	
Anthropometric		**Mean**	**Median**	**Range**
	**Age** (years)	35	34	24–49
	**Body weight** (kg)	66.6	65.0	44.0–87.0
	**Height** (cm)	162	164	146–173
	**Gestation weeks** (n)	27	26	24–34

* Student, domestic worker, performing military service or mandatory community service.

**Table 2:** Biomarkers of exposure to mycotoxins determined in urine and breast milk samples of the 19 mother–child pairs participating in the earlyMYCO pilot study. Results are presented as biomarker volume-weighted concentration (ng/mL).

			AFB_1_	OTA	DON	bZEL		AFB_1_	aZEL	FB_1_	FB_2_	FB_3_
		Moment of Observation (Number of Samples)	Urine				Moment of Observation (Number of Samples)	Breast Milk				
**Mother**	<LOD	**M1 (n = 19)**	19	17	19	19		-	-	-	-	-
	LOD < x < LOQ		0	0	0	0		-	-	-	-	-
	>LOQ		0	2	0	0		-	-	-	-	-
	Range (ng/mL)		-	3.67–4.43	-	-		-	-	-	-	-
	<LOD	**M2 (n = 9)**	9	9	7	8	**M2 (n = 7)**	5	5	5	5	7
	LOD < x < LOQ		0	0	2	1		0	0	0	2	0
	>LOQ		0	0	0	0		2	2	2	0	0
	Range (ng/mL)		-	-	-	-		0.44–0.47	8.67–9.66	10.0–10.1	-	-
	<LOD	**M3 (n = 8)**	8	8	8	7	**M3 (n = 4)**	3	4	3	3	3
	LOD < x < LOQ		0	0	0	1		1	0	1	1	1
	>LOQ		0	0	0	0		0	0	0	0	0
	Range (ng/mL)		-	-	-	-		-	-	-	-	-
**Child**	<LOD	**M2 (n = 9)**	8	8	7	9		-	-	-	-	-
	LOD < x < LOQ		1	1	0	0		-	-	-	-	-
	>LOQ		0	0	2	0		-	-	-	-	-
	Range (ng/mL)		-	-	8.35–44.07	-		-	-	-	-	-
	<LOD	**M3 (n = 7)**	7	7	5	7		-	-	-	-	-
	LOD < x < LOQ		0	0	1	0		-	-	-	-	-
	>LOQ		0	1	1	0		-	-	-	-	-
	Range (ng/mL)		-	21.29	25.63	-		-	-	-	-	-

AFB_1_ = Aflatoxin B_1_; DON = Deoxynivalenol; aZEL = alpha-zearalenol; bZEL = beta-zearalenol; OTA = ochratoxin A; FB_1_ = fumonisin B_1_; FB_2_ = fumonisin B_2_; FB_3_ = fumonisin B_3_; LOD = Limit of Detection; LOQ = Limit of Quantification; M1 = 1st moment of observation; M2 = 2nd moment of observation; M3 = 3rd moment of observation.

**Table 3:** Food consumption data obtained through a food frequency questionnaire and 24 h dietary recall.

	Food Frequency Questionnaire						24 h Recall					
	M1 (n = 19)						M1 (n = 19)		M2 (n = 9)		M3 (n = 4)	
	Never	1–3 Day/Month (n)	1 Day/Week (n)	2–3 Days/Week (n)	4–5 Days/Week (n)	6–7 Days/Week (n)	Median (g/Day)	IQR (g/Day)	Median (g/Day)	IQR (g/Day)	Median (g/Day)	IQR (g/Day)
Bread												
White bread	3	1	3	1	0	11	87.5	62.8–200.8	172.0	113.3–196.0	109.5	-
brown bread	2	3	5	5	1	3						
Cereals												
Breakfast cereals	3	5	4	6	0	1	30.0	30.0–34.0	91.0	-	NR	-
Cookies	1	4	4	7	1	2	131.0	50.0–260.0	78.5	46.8–110.3	56.0	-
Cakes	1	4	6	3	2	3						-
Rice	15	0	1	2	0	1	78.3	42.6–143.0	143.0	-	NR	-
Pasta	0	1	2	5	7	4	102.4	81.7–174.3	94	-	NR	-
Chocolate	1	4	4	3	2	5	NR	NR	NR	NR	NR	-
Nuts and seeds	5	7	1	1	1	4	170.0	-	20.0	17.0–24.0	NR	-
Dairy products												
Milk	5	1	0	1	0	12	175.2	83.9–312	312.0	275.0–392.0	503.0	-
Cheese	0	0	0	6	4	8	64.0	31.0–83.5	26.0	23.0–77.5	63.0	-
Yogurt	3	3	3	3	0	7	NR	NR	NR	NR	NR	-
Non-alcoholic drinks												
Coffee	5	0	0	1	0	13	58.0	40.0–170.5	34.5	31.2–40.0	40.0	-
Tea	1	2	5	4	1	6	312.0	-	NR	NR	NR	-
Animal products												
Red meat	2	1	1	10	4	1	165.5	111.3–190.1	173	-	58.0	-
White meat	2	0	1	10	5	1	80.3	63.7–108.0	126	-	114.0	-
Processed meat	5	4	4	4	1	1	18.0	10.0–23.0	23.0	-	NR	-
Eggs	1	5	0	6	3	3	160.0	-	57	-	NR	-
Fat fish	4	7	3	4	1	0	115.5	84.0–373.4	93.5	56.8–130.3	142.0	-
Predatory fish	13	2	1	3	0	0						-
Molluscs	5	10	3	1	0	0	44.1	-	20.0	-	NR	-

M1 = 1st moment of observation; M2 = 2nd moment of observation; M3 = 3rd moment of observation; IQR = Interquartile range; NR = Not Reported.

## Data Availability

Not applicable.

## Supplementary Materials

The following supporting information can be downloaded at: https://www.mdpi.com/article/10.3390/ijerph19137716/s1, Table S1: Main characteristics of published studies considered for designing the earlyMYCO pilot study. Table S2: Optimized MRM parameters for mycotoxins biomarkers (n = 18) and internal standards (n = 3) in urine and breastmilk. Table S3: Performance characteristics of the analytical LC-MS/MS method for urine samples. Table S4: Performance characteristics of the analytical LC-MS/MS method for breastmilk samples. Table S5: Questionnaires and biological samples (n) collected under the earlyMYCO pilot study.
