# Supplementary material for: earlyMYCO: A Pilot Mother-Child Cohort Study to Assess Early-Life Exposure to Mycotoxins—Challenges and Lessons Learned
Source: Int J Environ Res Public Health. 2022 Jun 23;19(13):7716. doi: 10.3390/ijerph19137716 (PMC9265400; doi:10.3390/ijerph19137716)
Supplement: Supplementary file 1 [file ijerph-19-07716-s001.zip › Supplementary Material.pdf]

Supplementary Material of the manuscript: earlyMYCO – a pilot mother-child cohort study to assess ear-ly-life exposure to mycotoxins

Table S1. Main characteristics of published studies considered for designing the earlyMYCO pilot study.

Reference	Study design	Participants	Moments of observation	Data collection	Biological samples	Analysis of environmental contaminants (Yes/No)
Galvano et al. (2008)	Cross-sectional	Women	Day 30 after birth	dietary questionnaire	Breast milk	Yes
Wright et al. (2013)	Cohort	Parents and children	26-28 weeks gestation<br>6, 12 and 18 months and 2, 3 and 4 years, after birth	sociodemographic, ethnicity and family trees, lifestyle factors, anthropometric measures, health data	Mother: blood, urine<br>Father: saliva<br>Children: cord blood, blood	Yes
Arbuckle et al. (2013)	Cohort	Women and children	Gestation: 6-13 weeks, 16-21 weeks, 32-34 weeks<br>Delivery<br>1-2 days and 2-10 weeks after birth	Sociodemographic, lifestyle (e.g., smoking and alcohol), medical history, use of natural health products and medications, and potential sources of exposure data, dietary questionnaire	Mother: blood, urine, breast milk<br>Children: cord blood, meconium	Yes

Heude et al. (2016)	Cohort	Parents and children	Mothers: 24-28 weeks gestation, delivery, 5-6 weeks after delivery<br>Children: birth, 1 year, 3 years, 5-6 years, 8 years	Health (e.g., anthropometric measures, blood pressure, infectious disease, mental health) Exposures (e.g., smoking, alcohol consumption, dietary behaviour, outdoor air pollution, indoor contaminants)<br>Behaviour and cognitive development (IQ, behaviour, other cognitive assessments)	Mother: blood, urine, hair, saliva, colostrum<br>Father: blood<br>Children: cord blood, meconium, hair, urine	Yes
Smith et al. (2017)	Cross-sectional	Women	Gestation: 14 weeks	demographics, dietary practices, childcare behaviours	Urine	Yes

Table S2. Optimized MRM parameters for mycotoxins biomarkers (n = 18) and internal standards (n = 3) in urine and breastmilk.

Analyte	Rt (min)	Q1 (mz^{-1})	CV (v)	Q3 (mz^{-1})	CE (v)	Dwell time (msec)	ESI-source
**AFB_1**	6.93	313	65	241 [a] /285	32/20	0.191	+
**AFB_2**	6.64	315	25	259 [a] /287	28/24	0.117	+
**AFG_1**	6.28	329	50	243 [a] /311	24/20	0.117	+
**AFG_2**	5.96	331	40	313 [a] /285	24/28	0.117	+
**AFM_1**	6.02	329	30	273 [a] /259	22/25	0.117	+
**OT-α**	5.91	257	30	239 [a] /221	10/20	0.117	+
**DON**	3.25	297	30	175 [a] /189	20/15	0.219	+
**aZEL**	9.33	321	30	175 [a] /177	22/17	0.119	+
**bZEL**	8.53	321	30	175 [a] /177	22/16	0.218	+
**FB_1**	8.28	722	40	352 [a] /344	32/36	0.218	+
**FB_2**	9.28	706	70	336 [a] /354	36/30	0.119	+
**FB_3**	10.37	706	70	354 [a] /530	30/28	0.608	+
**NIV**	2.30	313	35	175 [a] /177	21/16	0.219	+
**ZEN**	9.67	319	40	283 [a] /301	12/10	0.119	+
**OTA**	9.36	404	40	239 [a] /358	22/12	0.119	+
**HFB_1**	7.95	406	40	334 [a] /236	19/19	0.086	+
**AFL**	7.70	297	45	269 [a] /226	20/25	0.032	+
**STE**	10.13	325	40	281 [a] /310	24/30	0.049	+
**$^{13}C_{15}$ DON**	3.25	312	30	245 [a] /103	10/10	0.219	+
**$^{13}C_{17}$ AFB_1**	6.93	330	40	301 [a] /285	22/26	0.191	+
**$^{13}C_{20}$ OTA**	9.36	424	40	232 [a] /230	30/20	0.119	+

Rt = retention time. Q1 = parent ion. CV = cone voltage. Q3 = daughters ions. CE = collision energy. AFB_1 = aflatoxin B_1; AFB_2 = aflatoxin B_2; AFG_1 = Aflatoxin G_1; AFG_2 = aflatoxin G_2; AFM_1 = aflatoxin M_1; OT-α = Ochratoxine alpha; DON = deoxynivalenol; aZEL = alpha-zearalenol; bZEL = beta-zearalenol; FB_1 = fumonisin B_1 ; FB_2 = fumonisin B_2; FB_3 = fumonisin B_3; NIV = nivalenol; ZEN = zearalenone; OTA = ochratoxine A; HFB_1 = hydrolyzed fumonisin B_1; AFL = aflatoxicol; STE = sterigmatocystin; $^{13}C_{15}$-DON = isotope-labelled ($^{13}C_{15}$) deoxynivalenol; $^{13}C_{17}$-AFB_1 = isotope-labelled ($^{13}C_{17}$) aflatoxin B_1; $^{13}C_{20}$-OTA = isotope-labelled ($^{13}C_{20}$) ochratoxin A. [a] Quantifier transition.

Table S3. Performance characteristics of the analytical LC-MS/MS method for urine samples.

Analyte	Calibration range (ng/mL)	R^2	LOD (ng/mL)	LOQ (ng/mL)	Apparent Recovery (%)	RSD_r (%)	RSD_R (%)	U (%)
**AFB_1**	0.25-16.00	0.9731	1.19	2.38	90.88	14.09	15.84	20.88
**AFB_2**	0.25-16.00	0.9944	1.01	2.03	98.60	4.98	10.85	19.00
**AFG_1**	0.25-16.00	0.9752	2.08	4.16	105.90	11.48	13.72	16.10
**AFG_2**	0.25-16.00	0.9505	1.32	2.65	95.08	10.77	15.40	19.89
**AFM_1**	0.25-16.00	0.9524	0.38	0.77	94.26	11.95	12.76	17.02
**OT-α**	0.25-16.00	0.9703	1.18	2.37	103.70	4.99	4.86	11.87
**DON**	2.50-160.00	0.9930	5.77	11.53	100.40	4.74	6.39	15.57
**aZEL**	2.50-160.00	0.9842	9.76	26.91	100.86	7.55	8.64	18.96
**bZEL**	2.50-160.00	0.9737	12.43	24.87	81.87	2.25	1.60	7.70
**FB_1**	2.50-160.00	0.9697	23.19	46.39	98.51	3.04	8.29	10.23
**FB_2**	2.50-160.00	0.9989	7.61	15.22	98.30	7.35	7.85	9.40
**FB_3**	3.00-192.00	0.9922	7.75	15.49	93.93	6.11	9.03	9.56
**NIV**	2.50-160.00	0.9870	10.20	20.40	91.93	9.06	17.46	22.33
**ZEN**	2.50-160.00	0.9947	5.56	11.11	92.47	4.10	6.98	18.54
**OTA**	0.25-16.00	0.9972	0.73	1.43	102.16	2.75	2.61	11.54

LOD = Limit of detection. LOQ = Limit of quantification. RSDr = relative standard deviation intra-day precision. SDR = relative standard deviation inter-day precision. U = measurement uncertainty. AFB_1 = aflatoxin B_1; AFB_2 = aflatoxin B_2; AFG_1 = Aflatoxin G_1; AFG_2 = aflatoxin G_2; AFM_1 = aflatoxin M_1; OT-α = Ochratoxin alpha; DON = deoxynivalenol; aZEL = alpha-zearalenol; bZEL = beta-zearalenol; FB_1 = fumonisin B_1; FB_2 = fumonisin B_2; FB_3 = fumonisin B_3; NIV = nivalenol; ZEN = zearalenone; OTA = ochratoxin A.

Table S4. Performance characteristics of the analytical LC-MS/MS method for breastmilk samples.

Analyte	Calibration range (ng/mL)	R^2	LOD (ng/mL)	LOQ (ng/mL)	Apparent Recovery (%)	RSD_r (%)	RSD_R (%)	U (%)
**AFB_1**	0.125-8.00	0.9998	0.20	0.39	95.33	3.26	1.28	12.86
**AFB_2**	0.125-8.00	0.9919	0.37	0.75	91.00	4.88	7.71	13.26
**AFG_1**	0.125-8.00	0.9990	0.31	0.62	96.00	0.71	3.80	9.87
**AFG_2**	0.125-8.00	0.9996	0.23	0.46	91.67	8.23	6.66	19.48
**AFM_1**	0.125-8.00	0.9990	0.38	0.77	90.67	4.37	15.35	12.86
**OT-α**	0.125-8.00	0.9987	0.47	0.95	100.67	2.56	0.92	7.57
**DON**	1.25-80.00	0.9930	3.01	6.02	100.20	6.43	0.13	17.30
**aZEL**	1.25-80.00	0.9920	3.00	6.00	115.33	5.12	4.13	15.61
**bZEL**	1.25-80.00	0.9958	2.70	5.40	118.43	3.63	3.01	14.52
**FB_1**	1.25-80.00	0.9989	3.40	6.80	98.63	2.13	4.75	12.79
**FB_2**	1.25-80.00	0.9998	1.99	3.98	115.53	1.33	0.34	3.86
**FB_3**	1.50-96.00	0.9997	2.38	4.76	114.97	5.53	1.68	12.10
**NIV**	1.25-80.00	0.9954	2.78	5.57	88.27	5.07	5.36	17.63
**ZEN**	1.25-80.00	0.9981	2.19	4.38	110.4	1.15	0.71	13.66
**OTA**	0.125-8.00	0.9987	0.66	1.32	118.00	5.71	0.71	11.31
**HFB_1**	0.625-40.00	0.9972	2.42	4.85	86.67	5.81	2.85	18.48
**AFL**	0.125-8.00	0.9945	0.27	0.55	95.67	12.86	6.15	18.48
**STE**	0.125-8.00	0.9941	0.77	1.54	108.67	5.66	2.77	18.46

LOD = Limit of detection. LOQ = Limit of quantification. RSDr = relative standard deviation intra-day precision. SDR = relative standard deviation inter-day precision. U = measurement uncertainty. AFB_1 = aflatoxin B_1; AFB_2 = aflatoxin B_2; AFG_1 = Aflatoxin G_1; AFG_2 = aflatoxin G_2; AFM_1 = aflatoxin M_1; OT-α = Ochratoxin alpha; DON = deoxynivalenol; aZEL = alpha-zearalenol; bZEL = beta-zearalenol; FB_1 = fumonisin B_1; FB_2 = fumonisin B_2; FB_3 = fumonisin B_3; NIV = nivalenol; ZEN = zearalenone; OTA = ochratoxin A; HFB_1 = hydrolyzed fumonisin B_1; AFL = aflatoxicol; STE = sterigmatocystin.

Table S5. Questionnaires and biological samples (n) collected under the earlyMYCO pilot study.

	Participant	24-28 weeks pregnancy			1st month			6th month		
		Interview based			Self-report based					
		Participants (n)	Missing (n)	Incomplete (n)	Participants (n)	Missing (n)	Incomplete (n)	Participants (n)	Missing (n)	Incomplete (n)
**Questionnaires**										
Sociodemographic	Mother	19	0	0	Nc	Nc	Nc	Nc	Nc	Nc
	Child	Nc	Nc	Nc	Nc	Nc	Nc	Nc	Nc	Nc
Food Frequency	Mother	19	0	0	Nc	Nc	Nc	Nc	Nc	Nc
	Child	Nc	Nc	Nc	Nc	Nc	Nc	Nc	Nc	Nc
24h Dietary Recall	Mother	19	0	0	9	0	0	8	3	1
	Child	Nc	Nc	Nc	9	0	0	8	3	1
**Biological Samples**										
Spot Urine	Mother	19	0	0	9	0	NA	8	1	NA
	Child	Nc	Nc	Nc	9	0	NA	8	1	NA
Blood	Mother	18	1	NA	Nc	Nc	Nc	Nc	Nc	Nc
	Child	Nc	Nc	Nc	Nc	Nc	Nc	Nc	Nc	Nc
Breast milk	Mother	Nc	Nc	Nc	9 (7+2*)	0	NA	8 (4+2*)	2	NA
	Child	Nc	Nc	Nc	Nc	Nc	Nc	Nc	Nc	Nc

Nc = No considered for this moment of observation; NA = Not applicable; *two children were fed with infant formula
